# Supplementary material for: Transcriptome Analysis of the Seed Shattering Mechanism in Psathyrostachys juncea Using Full-Length Transcriptome Sequencing
Source: Plants (Basel). 2024 Dec 11;13(24):3474. doi: 10.3390/plants13243474 (PMC11728615; doi:10.3390/plants13243474)
Supplement: Supplementary file 1 [file plants-13-03474-s001.zip › plants-3306482-supplementary.pdf]

Supplemental Table S1. Statistical analysis of sequencing data for samples.

Sample	Raw reads	Clean reads	Clean bases	Q20 (%)	Q30 (%)	GC content (%)
H7_1	63399806	62547880	9232903259	97.33	95.29	53.41
H7_2	54957010	54292788	8010118655	97.19	95.00	53.10
H7_3	50638214	50032786	7406936414	97.10	94.84	52.94
H14_1	58117022	57437730	8475190818	97.23	95.09	52.88
H14_2	45938840	45375140	6727081854	97.08	94.80	53.21
H14_3	54658932	54063230	7872973404	97.29	95.24	52.66
H21_1	61889320	61040928	8883706678	97.34	95.35	52.99
H21_2	48699660	48082726	7061487536	97.18	95.06	52.89
H21_3	42585666	41956422	6206092118	96.88	94.46	53.07
H28_1	57782414	57128962	8407019657	97.12	94.94	53.02
H28_2	47816292	47173252	6972199059	97.22	95.11	55.40
H28_3	45275132	44558098	6574275518	97.04	94.81	55.30

Supplemental Table S2. GO enrichment analysis and statistics of shared genes at different developmental stages in *Psathyrostachys juncea.*

GO ID	Term Type	Description	*P* value	Number
GO:0016998	BP	cell wall macromolecule catabolic process	1.92E-07	16
GO:0044347	BP	cell wall polysaccharide catabolic process	1.99E-07	11
GO:2000895	BP	hemicellulose catabolic process	1.99E-07	11
GO:0045493	BP	xylan catabolic process	1.99E-07	11
GO:0009749	BP	response to glucose	2.40E-07	7
GO:0010383	BP	cell wall polysaccharide metabolic process	2.76E-07	17
GO:0034284	BP	response to monosaccharide	2.92E-07	7
GO:0009746	BP	response to hexose	2.92E-07	7
GO:0010252	BP	auxin homeostasis	2.92E-07	6
GO:0044264	BP	cellular polysaccharide metabolic process	3.99E-07	33
GO:0010410	BP	hemicellulose metabolic process	4.27E-07	17
GO:0044262	BP	cellular carbohydrate metabolic process	4.79E-07	38
GO:0045491	BP	xylan metabolic process	5.19E-07	13
GO:0010279	MF	indole-3-acetic acid amido synthetase activity	4.72E-08	6
GO:0016682	MF	oxidoreductase activity, acting on diphenols and related substances as donors, oxygen as acceptor	1.06E-07	9
GO:0004097	MF	catechol oxidase activity	1.57E-07	6
GO:0016160	MF	amylase activity	1.99E-07	14
GO:0016684	MF	oxidoreductase activity, acting on peroxide as acceptor	2.12E-07	20
GO:0016161	MF	beta-amylase activity	2.20E-07	14
GO:0102229	MF	amylopectin maltohydrolase activity	2.20E-07	14

Supplemental Table S3. Detailed profile analysis.

	Gene ID	GO ID	KO name	Description
Profile 19	H28_3_transcript_111743	GO:0008171	ASMT	acetylserotonin O-methyltransferase
	H28_3_transcript_8397	GO:0016491	adh	aldose reductase
	H28_3_transcript_106938	GO:0006979	E1.11.1.7	peroxidase
	H28_3_transcript_85209	GO:0004497	---	indole-2-monooxygenase
	H28_3_transcript_89139	---	TGA	transcription factor TGA
	H28_3_transcript_10101	GO:0016998	CHIB	basic endochitinase
	H28_3_transcript_105184	GO:0045493	---	chitinase CLP
	H28_3_transcript_107564	GO:0009734	PIN	auxin efflux carrier component
	H28_3_transcript_84147	GO:0016763	---	xylan glycosyltransferase MUCI
	H28_3_transcript_6471	---	PXG	peroxygenase
Profile 0	H28_3_transcript_82356	GO:0016161	E3.2.1.2	beta-amylase
	H28_3_transcript_103579	GO:0030245	E3.2.1.4	endoglucanase
	H28_3_transcript_65747	GO:0102500	malQ	4-alpha-glucanotransferase DPE
	H28_3_transcript_83088	GO:0004564	SST	sucrose 1-fructosyltransferase
	H28_3_transcript_7959	GO:0003700	K09264	MADS-box transcription factor
	H28_3_transcript_64323	GO:0003677	---	MYB transcription factor
	H28_3_transcript_90591	GO:0071555	---	cellulose synthase
	H28_3_transcript_78063	GO:0047334	PFP	pyrophosphate--fructose 6-phosphate
	H28_3_transcript_94771	GO:0016021	GAE	UDP-glucuronate 4-epimerase
	H28_3_transcript_79828	GO:0019318	HK	hexokinase
	H28_3_transcript_106697	GO:0009664	---	expansin
Profile 18	H28_3_transcript_128523	GO:0016491	GA2ox	gibberellin 2-beta-dioxygenase
	H28_3_transcript_104517	GO:0016705	CYP92A6	trimethyltridecatetraene synthase
	H28_3_transcript_108593	GO:0003700	---	bZIP transcription factor
	H28_3_transcript_122172	GO:0000981	NFYB	nuclear transcription factor
	H28_3_transcript_93173	GO:0046983	---	ICE transcription factor
	H28_3_transcript_13927	GO:0008757	AAMT	O-methyltransferase
	H28_3_transcript_99186	GO:0003700	---	NAC domain-containing protein
	H28_3_transcript_86714	GO:0015145	STP	MST sugar transport protein
	H28_3_transcript_88945	GO:0004565	E3.2.1.21	beta-glucosidase
	H28_3_transcript_75395	GO:0010279	GH3	indole-3-acetic acid-amido synthetase
	H28_3_transcript_77280	GO:0007166	---	wall-associated receptor kinase

Supplemental Table S4. Details of the gene set enrichment analysis for "H28 vs H7".

Gene set name	Description	NES	*P* value	*P* adjust	Gene number
MAP00500	Starch and sucrose metabolism	-2.14	0.00	0.00	110
MAP00520	Amino sugar and nucleotide sugar metabolism	-1.88	0.00	0.00	53
MAP00051	Fructose and mannose metabolism	-1.89	0.00	0.00	26
MAP00940	Phenylpropanoid biosynthesis	1.55	0.01	0.08	33
MAP00073	Cutin, suberine and wax biosynthesis	1.42	0.03	0.15	40

Supplemental Table S5. *Psathyrostachys juncea* germplasm and sources.

**Accession ID**	**Origin**	**Cultivation**	**Number of plants used**
PI 502577	Russian Federation	Cultivate	1
PI 619487	Mongolia	Wild	1
PI 619565	Mongolia	Wild	1
PI 531827	Estonia	Wild	1
PI 502573	Former Soviet Union	Cultivate	3
PI 272136	Alma-Asa, Kazakhstan	Cultivate	2
PI 502576	Russian Federation	Cultivate	1

Supplemental Table S6. Primers were used for qRT-PCR analysis.

Gene name	Gene ID	primer (5’~3’)
INV	H28_3_transcript_83241	F: GCCGCTACGACTACTACACC<br>R: CGGTATCAACAAAGCCTGCG
HK	H28_3_transcript_10291	F: ACGACAAAGCGTTGGATTTCG<br>R: AGCTTGGCAACCAGTGAAGA
FRK	H28_3_transcript_117447	F: ATGTTCTACCGCAACCCCAG<br>R: CGAAGGGATTGCCCCTTTCT
BG	H28_3_transcript_85611	F: TACCAACTCAACTGCCGACC<br>R: GTACCGCTTGAGGGTTGTGA
SPS	H28_3_transcript_93623	F: GCTCGTGTGGAAAAACACCC<br>R: CCACCCATGCTCGGTACTTT
EG	H28_3_transcript_129189	F: TTGTCAGGTTACAGCAGGGAA<br>R: CACGATAACACGCAGCCAC
SUS	H28_3_transcript_1371	F: CGCTGGAGAAGTTCCTTGGT<br>R: GCCAACGGTGTCCTTGTTTC
MADS-box	H28_3_transcript_7959	F: TGGAGCACTGGAAGATGCAC<br>R: AGGTGAACGGCATTTGGGAA
POD	H28_3_transcript_106938	F: TGTATGGGCTTATGCGGTGG<br>R: ATCTGGACGAAGCAGTCGTG
PXG	H28_3_transcript_6471	F: CGGTTGGGTTCAACGTGTTC<br>R: TCGAAGTTCACCGGCATGAA
Actin	-	F: TGGTATGGAAGCTGCTGGAA<br>R: TCAGCAATACCCGGGAACAT
